# Levels, trends, causes, place and time of, care-seeking for, and barriers in preventing indirect maternal deaths in Bangladesh: An analysis of national-level household surveys

**DOI:** 10.7189/jogh.13.04019

**Published:** 2023-04-28

**Authors:** M Moinuddin Haider, Abu Bakkar Siddique, Sabrina Jabeen, Aniqa Tasnim Hossain, Shusmita Khan, Md Mahabubur Rahman, Fatema Tuz Zohora, Nitai Chakraborty, Quamrun Nahar, Ahmed Ehsanur Rahman, Kanta Jamil, Shams El Arifeen

**Affiliations:** 1 International Centre for Diarrhoeal Disease Research, Bangladesh, Dhaka, Bangladesh; 2 Data for Impact, University of North Carolina at Chapel Hill, Chapel Hill, North Carolina, USA; 3 Independent consultant

## Abstract

**Background:**

Eclampsia, haemorrhage, and other direct causes are the primary burden of maternal mortality in Bangladesh, often reducing attention given to indirect maternal deaths (IMDs). However, Sustainable Development Goals may not be achieved without actions to prevent IMDs. We examined the levels, trends, specific causes, timing, place, and care-seeking, and explored the barriers to IMD prevention.

**Methods:**

We used three nationally representative surveys conducted in 2001, 2010, and 2016 to examine levels and trends in IMDs. The analysis of specific causes, timing, and place of IMDs, and care-seeking before the deaths was based on 37 IMDs captured in the 2016 survey. Finally, we used thematic content analysis of the open history from the 2016 survey verbal autopsy (VA) questionnaire to explore barriers to IMD prevention.

**Results:**

After increasing from 51 deaths per 100 000 live births in 2001 to 71 in 2010, the indirect maternal mortality ratio (IMMR) dropped to 38 deaths per 100 000 live births in 2016. In 2016, the indirect causes shared one-fifth of the maternal deaths in Bangladesh. Stroke, cancer, heart disease, and asthma accounted for 80% of the IMDs. IMDs were concentrated in the first trimester of pregnancy (27%) and day 8-42 after delivery (32%). Public health facilities were the main places for care-seeking (48%) and death (49%). Thirty-four (92%) women who died from IMDs sought care from a health facility at least once during their terminal illness. However, most women experienced at least one of the “three delays” of health care. Other barriers were financial insolvency, care-seeking from unqualified providers, lack of health counselling, and the tendency of health facilities to avoid responsibilities.

**Conclusions:**

IMMR remained unchanged at a high level during the last two decades. The high concentration of IMDs in pregnancy and the large share due to chronic health conditions indicate the need for preconception health check-ups. Awareness of maternal complications, proper care-seeking, and healthy reproductive practices may benefit. Improving regular and emergency maternal service readiness is essential.

Maternal deaths refer to female deaths during pregnancy and within 42 days of the end of pregnancy, irrespective of pregnancy duration and sites, place of death, and cause of death, except accidental or incidental deaths [1,2]. The deaths are broadly classified as direct maternal death (DMD) if directly linked to pregnancy or childbirth [1,3], and indirect maternal death (IMD) if due to pre-existing or newly developed health problems unrelated to pregnancy but which are aggravated by the effects of pregnancy [1,3]. The common causes of DMDs are eclampsia and haemorrhage [4]. On the other hand, IMDs are diverse, including deaths due to infections (e.g. sepsis), non-communicable diseases (NCDs) (e.g. heart disease), and other health conditions (e.g. anaemia) [3,4].

The distribution of DMD and IMD is not similar in all populations. In general, DMDs are the primary burden in the low- and middle-income countries; and IMDs in the high-income countries (HICs) [5-7]. These differences introduce the concept of “obstetric transition,” especially in HICs because the high rate of DMDs has been replaced by increasing rates of IMDs [8]. Many developed countries, like Sweden and England, started the transition more than a century ago [8,9], and a few developing countries, like Sri Lanka, in recent years [10]. By contrast, China is an exceptional example: maternal mortality is low, as in HICs (29 per 100 000 live births); nevertheless, postpartum haemorrhage is the primary cause of maternal deaths, a common phenomenon in a high maternal mortality population [11].

Globally, a total of 293 000 maternal deaths were estimated in 2013, of which 33 000 were due to indirect causes [12]. In the previous 23 years (1990-2013), the maternal mortality ratio (MMR) declined from 25 to 12 per 100 000 live births in developed countries. A declining trend was also seen in developing countries; nevertheless, the MMRs were high, between 233 and 318 per 100 000 live births [4]. The MMRs were between 252 and 383 per 100 000 live births in South Asia, with a higher ratio in neighbouring India and Pakistan, than in Bangladesh [12]. IMDs accounted for approximately one in four of the total maternal deaths in these countries [13-15].

The specific causes of IMDs vary widely across different populations. H1N1 influenza was a leading cause of IMDs in Brazil from 2009 to 2010 [16]; human immunodeficiency virus (HIV)/acquired immunodeficiency syndrome(AIDS) has been a leading cause in sub-Saharan Africa since the 1990s [17-19], and cardiac diseases in the United Kingdom [7]. Anaemia, diabetes, liver and kidney diseases, cancer, mental disorders, etc., are the other major causes of IMDs worldwide [13,14,16,20].

The MMR estimates in Bangladesh vary across sources. The Bangladesh Maternal Mortality and Health Care Survey series conducted by the Ministry of Health and Family Welfare is one of the most dependable sources for maternal mortality estimates. It showed that the MMR declined from 322 per 100 000 live births in 2001 to 194 per 100 000 live births in 2010 and remained unchanged (196 per 100 000 live births) until 2016 [15,21,22]. Like most other low- and middle-income countries, direct maternal mortality has historically been the primary burden in Bangladesh and an unfinished agenda until today. Although the maternal mortality prevention programs do not focus on IMDs [20], to move forward to achieve the health-related Sustainable Development Goals, prevention of IMDs is no less critical.

Studies on exploration, association or causation, and prevention of maternal mortality in Bangladesh in the last 50 years have mainly emphasized DMDs with minimum or no discussion about IMDs. Many studies were conducted at the sub-national level. Existing studies discussing IMDs mainly focused on levels of indirect MMR, the specific causes of IMDs, and the complexity of classifying IMDs [23,24]. For example, Chowdhury et al., estimated that indirect maternal mortality rate during 1996-2005 was 50 per 100 000 live births in a rural area [23]. However, to our knowledge, no study comprehensively explored the specific causes of IMDs, care-seeking behaviour, and the timing of IMDs at the national level to highlight the possible preventive measures, e.g. prompt care for sickness.

Although prompt care for a severe sickness saves lives, it does not happen always. Koenig et al. reported delays in recognizing, care-seeking, transportation, and care-receiving before maternal deaths [25]. However, what happened during the different stages of delays can exhaustively be understood from case studies that, to our knowledge, had not been documented in previous studies. Leaving these contexts behind is leaving many opportunities of preventing deaths undisclosed. This paper aims to study indirect maternal mortality in Bangladesh to highlight the missing opportunities to prevent IMDs.

## Study objectives

### Objective 1

Knowing the magnitude of a cause-specific mortality comes first to understanding its burden. So, we examined the levels and trends in indirect maternal mortality.

### Objective 2

When, where, and how the deaths happened are the essential questions to be answered for effective programmatic actions. Therefore, we explored the specific causes, timing, and place of, and care (care-seeking and receiving) for IMDs during terminal illness.

### Objective 3

Prompt care during a critical illness is essential to save lives. However, individual, social, and facility-level barriers often cause delays to arrange care for the sick person. So, we investigated the barriers to care-seeking and care-proving before death.

## METHODS

We used the nationally representative 2001, 2010, and 2016 Bangladesh Maternal Mortality Surveys (BMMSs). The surveys adopted the Demographic and Health Survey (DHS) Program’s Birth History Module to capture live births, and the Recent Household Death Module to record household deaths in the past three years [26]. Causes of all adult female (13-49 years) deaths were assessed using World Health Organization’s (WHO) Standard Verbal Autopsy (VA) tools. According to the International Classification of Diseases 10 (ICD 10), physicians trained in VA assessment assigned the causes of death. Following WHO guidelines, all deaths among females ages 13-49 years (addressed as only women/female in the following sections) that occurred during pregnancy or within 42 days of pregnancy outcome, except accidental or incidental deaths, were classified as maternal deaths [27]. The National Institute of Population Research and Training (NIPORT), in collaboration with the International Centre for Diarrhoeal Disease Research, Bangladesh (icddr,b) and MEASURE Evaluation in the United States conducted the BMMSs. Further details on the surveys, the identification of maternal deaths, and the assignment of causes of maternal deaths are available elsewhere [15,21,22]. **Table 1** shows the number of households and women interviewed, livebirths captured, and female deaths captured.

**Table 1:** Survey time, adult female deaths, and livebirths, 2001, 2010, and 2016 Bangladesh Maternal Mortality Surveys (BMMS)

	2001 BMMS	2010 BMMS	2016 BMMS
Data collection	January-June 2001	January-August 2010	August 2016-February 2017
Number of households interviewed (response rate)	99 202 (98.8%)	168 629 (98.4%)	298 284 (99.1%)
Number of ever-married women ages 13-49 years interviewed (response rate)	103 796 (97.2%)	175 621 (97.3%)	321 214 (95.6%)
Number of deaths captured among females ages 13-49 years	928	901	1524
Number of maternal deaths (13-49 years) in the three years preceding the survey (unweighted)	141	105	146
Number of live births among women (13-49 years) captured in the three years preceding the survey (unweighted)	40 478	52 051	81 043
Number of total maternal deaths captured	-	-	175
Number of maternal deaths due to indirect causes	-	-	37

BMMS – Bangladesh Maternal Mortality Survey

### Analysis

#### Analysis for objective 1

We used the MMR as the measure for examining the levels of maternal mortality. MMR was estimated as the ratio of the maternal mortality rate over the general fertility rate in the three years preceding the survey [15] and interpreted as maternal deaths per 100 000 live births. We estimated overall MMR and indirect MMR, and share of IMDs in the 2001, 2010, and 2016 rounds of the BMMS. The indirect MMRs are reported with error bars presenting 95% confidence intervals (CI). The CIs may be relatively wide due to the rarity of the pregnancy related death events (IMDs in our analysis).

#### Analysis for objective 2

The analysis of specific causes of IMDs, timing and place of death, and care-seeking during terminal illness relied on the 2016 BMMS only and results are described in numbers and percentages. The analysis of MMR in the three rounds of BMMS used a three-years period, that is, it included maternal deaths and live births in the three years prior to the survey. But, this analysis included all IMDs captured in the survey rather than limiting the analysis period to the past three years.

The 2016 BMMS used VA tools comparable with the 2012 WHO VA questionnaire version and assigned ICD 10 codes after assessment. Further details on the VA assessment are available elsewhere [15]. First, we documented ICD10 codes for each of the 37 IMDs and summarized the specific causes of IMDs. We also report common health conditions that the deceased had, as reported by the VA respondents.

The categories of time of deaths were adopted from the Lancet paper by Ronsmans et al. with a little modification [28]. The categories in the Lancet paper were during pregnancy, day 1, day 2, day 3-7, day 8-42, day 43-90, day 91-180, day 181-365, years 2. We divided the pregnancy period into first, second, or third trimesters; labour to delivery and day of delivery as day 1, day 2, day 3-7, and day 8-42. The remaining categories used by Ronsmans et al. have been excluded as this paper considered pregnancy to 42^nd^ day of delivery as the time of maternal death [28].

Places of deaths were home, and two types of health facilities – public health facility, private health facility, and in-transit (on the way from home to a facility or from facility to facility). These categories suit the best in the Bangladesh context, and have been used in a few unpublished studies on overall maternal mortality, maternal mortality due to hemorrhage and eclampsia.

We also examined the times and places of care-seeking at the terminal illness by every care-seeking instance, and summarized the first care facility, last care facility, multiple care facilities, times care was sought, and sequence of facilities or providers for multiple care-seeking.

We present the levels, causes, place, and care-seeking statistics in percentages, although in most cases, we did not interpret the findings using percentages, rather, we focused on the pattern by characteristics. Because the number of IMDs was small (n = 37), we used this approach to avoid misleading conclusions. We suggest that readers use the small denominator-based percentages with care.

#### Analysis for objective 3

We conducted a thematic analysis and quantified the themes.

#### Thematic analysis

The 2016 BMMS did not collect qualitative data using in-depth interviews or other qualitative data collection methods to understand the deep-rooted supply and demand-side barriers for preventing maternal deaths. However, the open history section of the VA questionnaire narrated by the respondents (who were close to the deceased woman during her last days) provided an opportunity to investigate barriers to the prevention of maternal deaths. We examined the VA open histories to identify important themes that described potential barriers in care-seeking by and care-providing to the women during terminal illness. We conducted a thematic analysis of the open history. Pseudo names are used in open history analyses.

#### Quantifying themes by content analysis

We also provide the proportion of cases where a specific theme was reported. This quantification includes single, combined, and additional themes that we did not summarize in the thematic analysis.

## RESULTS

**Figure 1:**
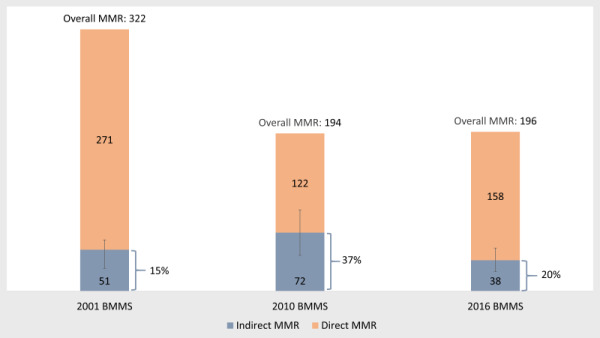
Maternal mortality ratios (MMR) in 2001, 2010 and 2016 Bangladesh Maternal Mortality Surveys (BMMSs), by causes.

Indirect MMR in 2001, 2010, and 2016 are presented in **Figure 1**. The 2016 BMMS estimated an IMMR of 38 per 100 000 live births. Although the overall MMR remained unchanged between 2010 (n = 194) and 2016 (n = 196), the IMMR dropped by 47%, from 72 in 2010 to 38 in 2016. As a result, the share of maternal deaths due to indirect causes declined from 37% to 20%. In 2001, the IMMR and the proportion of IMDs were close to the estimates obtained in 2016.

**Figure 2:**
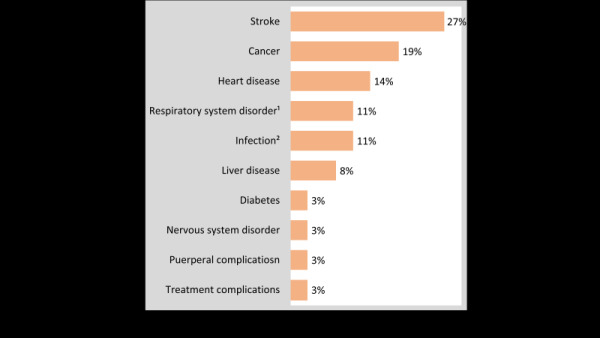
Percentage distribution of causes of indirect maternal deaths (IMDs), n = 37. Note: ^1^three of four were asthma, ^2^typhoid, tetanus, sepsis, and others.

**Figure 2** shows the specific causes of IMDs. Stroke, cancer and heart diseases caused 60% of IMDs (27%, 19% and 14%, respectively). The other 40% of the IMDs were due to diverse causes like infections and NCDs. ICD10 codes for each of the IMDs are documented in Table S1 in the **Online Supplementary Document**.

Women died of indirect maternal causes had several chronic health conditions, acute illness, and other severe physical discomforts (data not shown). History of asthmatic conditions and high blood pressure was reasonably common among the women who died of indirect maternal causes. Chronic kidney and liver problems and health problems related to a previous pregnancy or childbirth were also reported. The deceased women commonly reported headache, breathing difficulty, fever, cough, abdomen and chest pain, excess vomiting, unusual sweating, irritability and instability, blood in the urine and vomiting, oedema, weakness, and blurred vision during terminal illness. Family members observed yellowish and pale eyes, face, and skin on the hands in a few cases.

**Figure 3:**
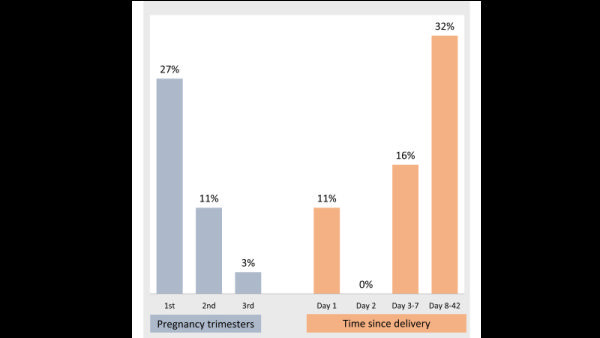
Percentage distribution of the timing of indirect maternal deaths (IMDs), 2016 Bangladesh Maternal Mortality Surveys (BMMS), n = 37.

**Figure 3** presents the timing of IMDs. Seventy percent of the IMDs occurred in the first trimester of pregnancy (27%) and day 8-42 after birth (32%).

**Figure 4:**
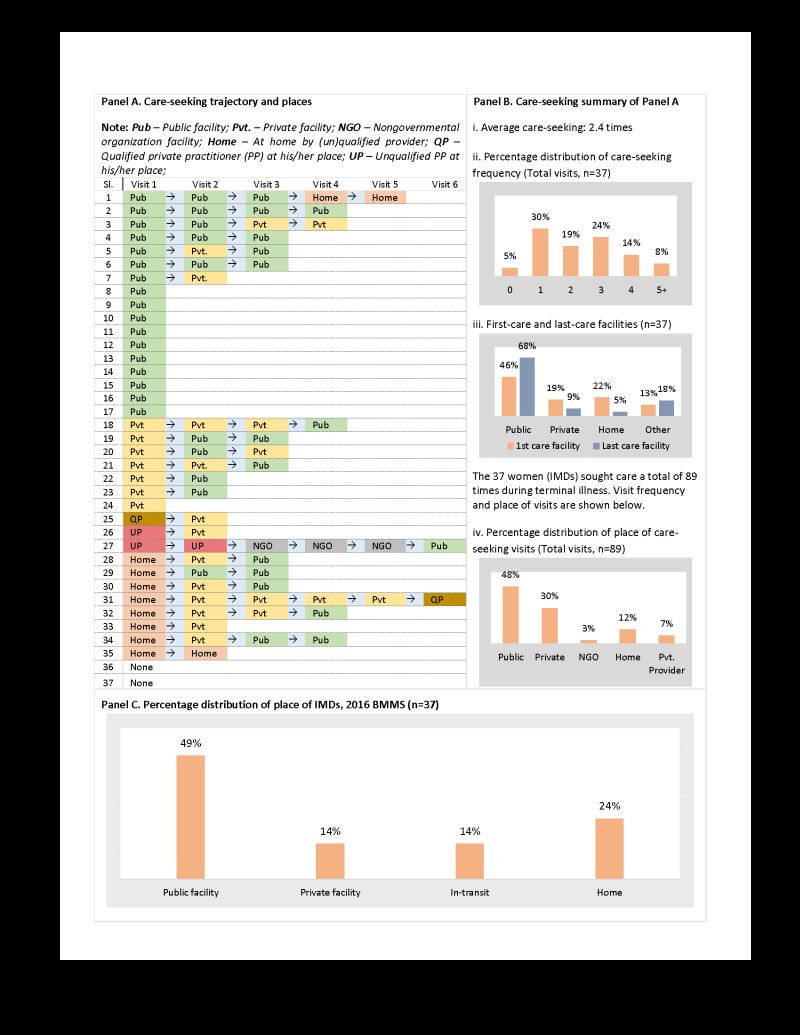
Place of care-seeking and death of indirect maternal deaths (IMDs), 2016 Bangladesh Maternal Mortality Surveys (BMMS). **Panel A**. Care-seeking trajectory and places. **Panel B**i. Average care-seeking. **Panel B**ii. Percentage distribution of care seeking frequency. **Panel B**iii. First-care and last-care facilities. **Panel B**iv. Percentage distribution of place of care-seeking visits. **Panel C**. Percentage distribution of place of IMDs.

Care-seeking place and frequency during terminal illness, and place of death are shown in **Figure 4**. Most women who died of indirect causes of maternal death (34 of 37) sought care from a health facility or a provider at least once (**Figure 4**, panel A). On average, deceased women sought care 2.4 times during terminal illnesses (**Figure 4****,** panel B.i), and care-seeking ranged up to six times (**Figure 4**, panel A). Near half (46%) of the women had to care-seek from more than two facilities (**Figure 4****,** panel B.ii). Care-seeking from a public health facility was much higher than from a private health facility (**Figure 4**, panel B.iii and panel B.iv). It was similar for dying. Half (18 of 37) of the women died at public facilities, 5 of 37 (14%) at private facilities, and 5 of 37 (14%) in-transit. Around one-quarter (9 of 37) died at home (**Figure 4****,** panel C).

### Barriers to preventing IMDs

#### Story 1. Tendency of hospitals / clinics to avoid taking on responsibilities

In her eighth month of pregnancy, Salima was taken to a private clinic in the afternoon with multiple complications, after receiving an initial care at home. Ultrasonography revealed that the child had died in the uterus. Although she needed an immediate caesarean operation, the clinic referred her to a public medical college hospital. After reaching there in the evening, she underwent several diagnostic tests and stayed in the facility for around 15 hours before being referred to a private hospital in the morning. At noon, she reached the facility and underwent a caesarean operation after nine hours of waiting. Unconscious, Salima was taken to the intensive care unit, where she died at 2 am. She was at or near a doctor's care for more than 25 hours, but she died, spending her most precious hours at health facilities waiting for essential care.

*Salima was taken to a private clinic. She underwent ultrasonography, and a stillborn baby was diagnosed in her uterus. They referred her to a Public Medical College Hospital. Salima reached the facility in the evening. Many diagnostic tests were done there. She was then referred to a private medical centre the next morning. She was taken to the private medical centre at 12 pm A caesarean operation was done to remove the stillborn baby at 9 pm Salima was unconscious in the intensive care unit. She died there at 2 am*

#### Story 2. The three delays

An untrained provider recommended to take unwell and nine months pregnant Rasima to a qualified doctor. But, in her husband's absence, family members could not decide what to do. Being informed, her husband came home and they left for a private clinic at 12 am. They reached the clinic at 3 am. The duty doctor said that she had severe anaemia and needed an immediate blood infusion, but the clinic did not have any blood. The doctor referred her to the district hospital. She was immediately taken to the district hospital, where the duty nurse gave her intravenous saline. Rasima died in the morning while waiting for a doctor.

*Because Rasima’s husband was not home, nobody could decide what to do.*

*They started for ABC private clinic at 12 am and reached there at 3 am.*

*She was admitted to the district hospital. A duty nurse gave her intravenous saline. Because it was midnight, no doctor was available.*

Stories on care-seeking at home and from unqualified providers, financial barrier to care-seeking, lack of health counseling and influence of national political issues on health services at facilities are documented in Story S1-S4 in the **Online Supplementary Document**.

**Figure 5:**
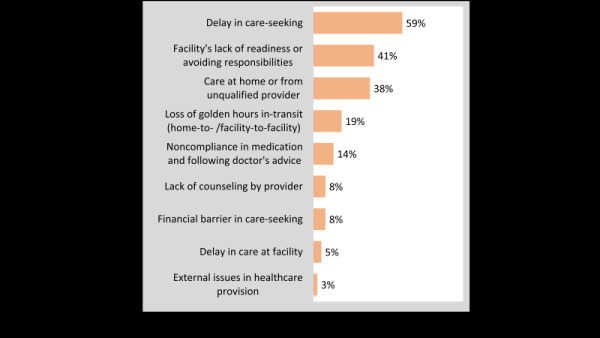
Barriers narrated by the respondents in the verbal autopsy (VA) open history (%), n = 37. Note: Themes are explained in Table S2 in the **Online Supplementary Document**.

Burden of the barriers presented in **Figure 5** shows the different obstacles that influenced the prevention of IMDs, and magnitudes of these various barriers. The commonest barriers were delays in appropriate care-seeking in six of 10 (59%) cases, tendency of health providers to avoid taking on responsibilities or lack of facility readiness (four of 10), and home care or care from an unqualified provider (four of 10). One in five women had to move from one facility to another at the time of their terminal illness when every moment makes a difference between life and death.

## DISCUSSION

The notable decline in DMDs (mainly deaths due to eclampsia and haemorrhage) helped reduce MMR from 332 per 100 000 live births in 2001 to 194 in 2010, which remained 196 in 2016 [21,22]. However, indirect MMR dropped slightly during the period – it increased from 51 per 100 000 live births in 2001 to 72 in 2010, then dropped to 38 in 2016. Such fluctuation in indirect maternal mortality rates observed in Matlab health and demographic surveillance system during 1976-2005 indicates it is a natural phenomenon [23]. An increase in ANC use and facility births during the 2000s were likely to be the reasons that helped reduce eclampsia and haemorrhage deaths. There are also studies supporting this explanation that adequate ANC-seeking helps protect against eclampsia and haemorrhage deaths [29,30].

The achievement in reducing maternal mortality harvested through improved maternity care and facility delivery faded away due to inadequate emergency obstetric services. Multiple visits to multiple health facilities during terminal illness are likely to be outcomes of weak referral system, lack of readiness, de-shouldering responsibilities, etc. Unavailability of service guidelines, trained staff, equipment and supplies, and diagnostic tests for antenatal care (ANC) and delivery care caused failure to identify and care for complex and risky pregnancies and deliveries (**Table 2**) [31,32]. Thus, the opportunities to prevent both DMDs and IMDs created through the high use of ANC and facility deliveries were lost [33]. Improvements in direct maternal mortality during the 2000s, which plateaued in the 2010s, indicate that the country has successfully prevented low-risk maternal deaths by increasing awareness of maternal health and use of maternity care (e.g. ANC, facility birth). However, existing health service systems appear to be incapable of satisfactorily addressing the other health complications during the pregnancy and postpartum periods, which are not due to obstetric causes but are aggravated by the physiologic effects of pregnancy. Moreover, the health sector program’s action plan to prevent deaths due to these health conditions during the maternity period, that is, the IMDs, is not well articulated [34]. Development of such an action plan requires a complete exploration of the variety and nature of the causes of IMDs.

**Table 2:** Selected components of antenatal care (ANC) and delivery care services in health facilities, Bangladesh*

Service guidelines, trained providers, equipment and supplies, and diagnostic tests in the health facilities in Bangladesh	2014 BHFS (%)	2017 BHFS (%)
Service guidelines		
*ANC guidelines*	50	46
*Guidelines on basic or comprehensive emergency obstetric care*	27	12
Trained provider		
*Staff trained in ANC care at any time*	49	55
*Staff trained in delivery care at any time*	39	45
Equipment and supplies		
*Blood pressure apparatus*	87	86
*Emergency transport*	33	30
*Oxytocin*	34	31
*Magnesium sulphate*	22	14
*Skin disinfectant*	27	54
*Running water*	72	79
*Soap*	90	75
*Alcohol-based hand disinfectant*	50	40
Diagnostic testing capacity		
*Urine glucose*	18	22
*Urine protein*	19	25
*Haemoglobin*	12	17

BHFS – Bangladesh Health facility Survey, ANC – antenatal care

*Data sources: [31,32].

This study identified that stroke, cancer, heart disease, and chronic asthma accounted for 80% of the IMDs in Bangladesh. The diseases were likely to develop before pregnancy, not induced by pregnancy. This means that preconception health conditions induced a large share of the IMDs. Health services (e.g. routine screening) for cancer, stroke, and heart disease-related health conditions are limited in routine setting and may become more cumbersome during the maternal period. Besides the care during pregnancy and pre-pregnancy stages, actions for preventing NCDs and their risk factors have to be integrated to maternal health strategies.

**Figure 6:**
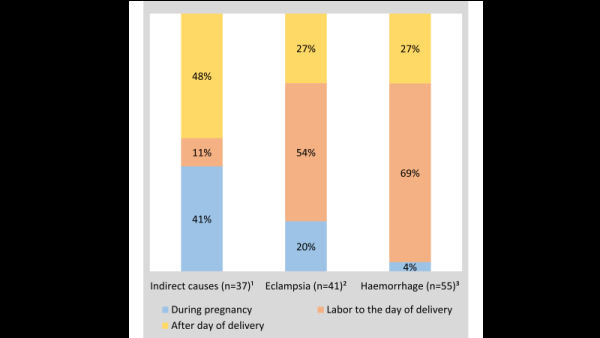
Percentage distribution of the timing of maternal deaths due to indirect causes, eclampsia, and haemorrhage, 2016 Bangladesh Maternal Mortality Surveys (BMMS). Note: the superscripts provide data source. ^1^**Figure 3** of this paper, ^2^(unpublished data), ^3^(unpublished data).

The comparison of the timing of hemorrhage, eclampsia, and IMDs also points to preconception health conditions (**Figure 6**). Forty-one percent of IMDs happened during pregnancy, with a higher frequency in the first trimester than in second and third trimesters. By contrast, 20% of the eclampsia deaths (unpublished data) and nearly none of the haemorrhage deaths happened during pregnancy (unpublished data). This means that preconception health conditions were aggravated immediately after conception. The provision and practice of preconception health check-ups could inform a woman and her family members about pregnancy decision making and appropriate care-seeking during the maternal period. An example is hypertension, which is an important determinant of stroke and heart disease. Around 21% of women ages 18-49 years are hypertensive, and about 10% of the women in this age group give birth in a year [35,36]. Therefore, a preconception check-up would be helpful to the couple for decision making and appropriate care-seeking behaviour during the pregnancy and postnatal periods. WHO recommends the provision and practice of preconception care, including medical and behaviour interventions [37,38].

We recommend the health service systems to focus on functioning referral systems, facility readiness, provider’s accountability, preconception medical care, and behavioural change communications to manage health conditions due to obstetric and aggravated by obstetric reasons. However, the efforts will go in vain without timely care-seeking.

Three in five women had delays in care-seeking. The delays may have resulted from a lack of community awareness about immediate and necessary actions to address pregnancy and postpartum complications. Although ANC from a medically trained provider is high, lack of counselling left women unaware of pregnancy danger signs and prompt care-seeking [34,35,39]. Knowledge may be even lower among men – the main household decision makers – because public health programs seldom include men in their interventions. Financial barriers and other socio-cultural obstacles, like gender-based neglect in health that starts immediately after a child is born, may have influenced the delayed care-seeking for health complications during maternity [40].

An improved care-seeking behaviour will not be sufficient preventing IMDs. Although delayed in many cases, most women who died of indirect maternal causes sought care from a place outside home, mainly from health facilities. No care or delayed care at the facilities forced the family to go from one facility to another for appropriate services, either being referred by the health facility or through self-initiative. The life-saving golden hours were lost on the road, and care-seeking from health facilities due to poor referral systems. [41,42]. Moreover, a referral should accompany an emergency transportation service which is rarely available (**Table 2**) [31,32]. These realities are true for both public and private sector health facilities.

For many years, private health facilities have been the main choice for ANC and birth care-seeking over public. However, public facilities were the primary place of care seeking during terminal illness and IMDs, which was similar for deaths due haemorrhage and eclampsia (unpublished data). As discussed above, the lack of facility readiness may have caused the referrals of terminally ill mothers from private to public health facilities. Moreover, incidences of violence against physicians and other atrocities after unpleasant events (e.g. death) may drive private and sub-district-level health facilities to avoid treating complicated and terminally ill patients and refer them elsewhere, mainly to upper-level public health facilities [43,44]. Concern about business reputation may be another reason that terminally ill patients are inappropriately referred from private to public health facilities.

### Strengths and limitations

The BMMS used the most reliable tools (DHS Birth History Module and Recent Household Death Module) to capture births and female deaths in a population-based study [26]. Therefore, minimal limitations associated with the tools and measurement methods, such as recall bias, misclassification, etc., affected the analysis. The sample size of the 2016 BMMS was statistically sufficient to estimate MMR but not sufficient for sub-group analysis. IMDs comprise a small part of overall maternal deaths (2001 BMMS: one in seven; 2010 BMMS: one in three; 2016 BMMS: one in five). As the analysis of levels, correlates, timing, and causes of IMDs was based on these small numbers, we do not make any strong conclusion and advise readers to interpret the findings with care.

In identifying the specific causes of IMDs, the study also suffered from the inherent limitations of VAs and physician-assessed causes of death. However, using VA, physician-assessed cause of death remains reliable [45]. Moreover, misclassification between direct and indirect maternal death is expected to be very negligible. In particular, in most cases, people dying from NCDs get medical assessments that are also used to assess the cause of death and assign ICD codes. A large share of the IMDs in this study was due to NCDs; therefore, we expect a minimum level of misclassification.

## CONCLUSIONS

One in five maternal deaths due to indirect causes makes it the third leading cause of maternal deaths in Bangladesh. The specific causes of IMDs are mainly chronic diseases and health conditions which previously existed. The IMDs due to chronic diseases and the large share of the IMDs in the first trimester and after the first week of the end of the pregnancy alert for initiating preconception care and improving necessary medical counselling and care during antenatal and postnatal contacts.

## Additional material


Online Supplementary Document
