# Supplementary material for: Levels, trends, causes, place and time of, care-seeking for, and barriers in preventing indirect maternal deaths in Bangladesh: An analysis of national-level household surveys
Source: J Glob Health. 2023 Apr 28;13:04019. doi: 10.7189/jogh.13.04019 (PMC10363693; doi:10.7189/jogh.13.04019)
Supplement: Online Supplementary Document [file jogh-13-04019-s001.pdf]

ONLINE SUPPLEMENTARY DOCUMENT

Title: Patterns of and barriers to preventing indirect maternal mortality in Bangladesh: An analysis of national-level household surveys

Authors: M. Moinuddin Haider[1*](https://orcid.org/0000-0002-5568-1195), Abu Bakkar Siddique[1](https://orcid.org/0000-0002-1484-0823), Sabrina Jabeen[1](https://orcid.org/0000-0002-0231-2070), Aniqa Tasnim Hossain[1], Shusmita Khan[2](https://orcid.org/0000-0003-3342-6211), Md Mahabubur Rahman[1](https://orcid.org/0000-0002-3283-5163), Fatema Tuz Zohora[1](https://orcid.org/0000-0003-4599-9658), Nitai Chakraborty[2](https://orcid.org/0000-0002-4101-7920), Quamrun Nahar[1], Ahmed Ehsanur Rahman[1†](https://orcid.org/0000-0001-9216-1079), Kanta Jamil[3†], Shams El Arifeen[1†](https://orcid.org/0000-0002-5372-5932)

[1]International Centre for Diarrhoeal Disease Research, Bangladesh

[2]Data for Impact, University of North Carolina at Chapel Hill, Chapel Hill, USA

[3]IAP World Services, USA

[*]Joint first authorship.

[†]Joint senior authorship.

Table S1: Summary matrix of ICD-10 codes used to present the cause of indirect maternal death

**Sl. #**	**ICD-10 Code, 2010 edition**	**Title**	**Number of cases**
1	A01.0	Typhoid fever	1
2	A09.0	Other and unspecified gastroenteritis and colitis of infectious origin	1
3	A34	Obstetrical tetanus	1
4	A41.9	Sepsis, unspecified	1
5	C22.9	Malignant neoplasm of liver, not specified as primary or secondary	2
6	C50.9	Malignant neoplasm of breast	1
7	C95.9	Leukaemia of unspecified cell type	3
8	D43.2	Neoplasm of uncertain behaviour of brain, unspecified	1
9	E10	Type 1 diabetes mellitus	1
10	G98	Other disorders of nervous system not elsewhere classified	1
11	I21.9	ST elevation (STEMI) myocardial infarction involving other coronary artery of anterior wall	1
12	I46.9	Cardiac arrest, cause unspecified	1
13	I50.9	Heart failure, unspecified	2
14	I51.9	Heart disease, unspecified	1
15	I64	Stroke, not specified as haemorrhage or infarction	10
16	J46	Status asthmaticus	3
17	J98.9	Respiratory disorder, unspecified	1
18	K76.9	Liver disease, unspecified	2
19	O26.6	Liver disorders in pregnancy, childbirth and the puerperium	1
20	O90.9	Complication of the puerperium, unspecified	1
21	T80.9	Unspecified complication following infusion, transfusion and therapeutic injection	1

Table S2: A brief description of the reported barriers to indirect maternal death prevention by themes

Themes	Brief description
Delay in care-seeking	- Not immediately care-seeking after the onset of visible severe health complications, e.g., swollen body, breathing difficulty, excessively bleeding, high blood pressure, soring abscess, etc.<br>- The delays were from hours to days to weeks.<br>- The delays were due to not acknowledging/understanding the severity of the problem, the absence of the husband, home care by unqualified providers, financial hardship, etc.
Lack of facility readiness or avoiding responsibilities	- No ICU facility<br>- No blood infusion facility<br>- Unavailability of physicians<br>- Unavailability of emergency supplies (e.g., oxygen supply)<br>- Referring severely ill pregnant or postpartum women to other facility who needed immediate care<br>- Restrain care at facilities due to political agitation
Care at home or from unqualified provider	- Self-explanatory
Loss of golden hours in-transit (home-to-facility or facility-to-facility)	- Shuttling from one facility to another, either being referred or by self-initiative for appropriate care<br>- Some of the fatalities also happened while traveling from home to a facility
Non-compliance to medication and following doctor's advice	- Not abiding by the doctor's advice on how and when to take the medicines<br>- Was fully aware of the advice but was reluctant<br>- Did not understand the importance of the doctor's advice
Lack of counselling by provider	- Provider advised not to conceive after the first birth but did explain why<br>- Provider advised healthy practices but did not explain what might happen if not followed
Financial barrier in care-seeking	- Self-explanatory
Delay in care at facility	- Mainly due to the unavailability of health providers at the facility at any given time, particularly during the nighttime
External issues at health care provision	- See Story 6 in the "Barriers to preventing IMDs" sub-section in the "Results" section

Story S1. Care-seeking at home and from unqualified providers: Shipa became pregnant for the first time at the age of 37. She had persistent vomiting, difficulty eating food and weakness for around a month during the third and fourth months of pregnancy. The last ten days of her life were the worst when she could not eat food at all, stopped passing stool, and had aches in her chest. She was never taken to a hospital or any medical doctor. She was diagnosed with jaundice by an untrained provider at home, medicated as the provider prescribed, and took *kabiraji*[1] medicines. Beginning the day before her death, she had a bloated stomach. Shipa was behaving abnormally in the last few hours before her death. Her body was fully stiff while she died.

> *Verbatim.* "She was not taken to any trained doctor or any renowned hospital. She had chest ache a few days before her death."

Story S2. Financial barrier to care-seeking: Although living with chronic asthma, hypertension, being overweight, and having kidney-related pregnancy complications, Rubana had a safe birth at home. However, she had swollen feet and breathing difficulties on the second day after the birth. An untrained provider treated her at home and she felt well over the next two days. Thirteen days before her death, she was taken to an Upazila health complex (sub-district-level facility) because her whole body became swollen and she was having severe breathing difficulties. Doctors diagnosed kidney damage and liver problems and referred her to the district hospital. Due to financial constraints, Shipa and her family members returned home. Despite her poor health conditions, Shipa was doing all the household chores. After nine days, she became severely sick when preparing to go to bed. Before death, she had bubbles on her face and her body was sweating. A doctor who came to the home confirmed that her death was due to stroke.

> *Verbatim.* "Doctors at the Upazila health complex referred Shipa to the district hospital. Her family members could not take her to the district hospital due to financial hardship."

Story S3. Lack of health counselling by health providers: The doctor at the health facility, after Anamika's first birth nine years previously, advised her not to have any more children. We assume that the doctor identified medical conditions, making another pregnancy unsafe. But did Anamika understand how fatal the next pregnancy could be? She had the right to be informed why the doctor made this recommendation. The doctor had the duty to counsel Anamika on "why" and "how" she should have acted to avoid pregnancy in the future. But the doctor did not explain anything, except telling her to avoid any further pregnancy. Anamika did not realize that the next pregnancy would be fatal. She conceived again, received antenatal care (ANC) visits, and had no complications, except

[1] *Kabiraji* medicines are traditional medicines, generally processed and prescribed by traditional providers known as *Kabiraj*.

anaemia. She had a normal vaginal birth at home, but the baby was stillborn. She had heavy bleeding, fragile health, and was heartbroken. A heart attack took her life very soon after the birth.

Verbatim. “Anamika gave birth to her first child nine years ago. The doctor, after the birth, advised her not to have any more children. However, as far as I know, the doctor did not tell her why she should have avoided any more pregnancies.”

Story S4. Influence of national political issues on health services at facilities: Seven months pregnant, Kalpona was taken to a private hospital in Dhaka after being referred by an unqualified provider at her home and by qualified doctors at a private clinic in Chattogram. She had high blood pressure and convulsions at that time. According to the VA respondent, the doctors were looking confused and tensed. Eventually, they refused to provide treatment to Kalpona. She was then taken to a public medical college where she was assessed as hypertensive and hypoglycaemic, and she died there after a few minutes. The respondent added that it was the verdict day for one of the accused persons for war crimes in the 1971 liberation war in Bangladesh.

What made the facility so hesitant to provide treatment to a severely sick pregnant woman was not clear. Possibly there were protests outside, the city people were passing through nail biting hours and the health facility authority was in dilemma to keep the facility open for services (not in the VA open history).

Verbatim. “On that day, the verdict of Alauddin was expected to be turned out. For this, the doctors could not decide if they were providing health care services or not. Finally, they said they could not provide health services to her.”
